# The demographic features of fatigue in the general population worldwide: a systematic review and meta-analysis

**DOI:** 10.3389/fpubh.2023.1192121

**Published:** 2023-07-28

**Authors:** Ji-Hae Yoon, Na-Hyun Park, Ye-Eun Kang, Yo-Chan Ahn, Eun-Jung Lee, Chang-Gue Son

**Affiliations:** ^1^Research Center for CFS/ME, Daejeon Oriental Hospital of Daejeon University, Daejeon, Republic of Korea; ^2^Department of Health Service Management, Daejeon University, Daejeon, Republic of Korea; ^3^Department of Korean Rehabilitation Medicine, College of Korean Medicine, Daejeon University, Daejeon, Republic of Korea; ^4^Institute of Bioscience and Integrative Medicine, Daejeon University, Daejeon, Republic of Korea

**Keywords:** fatigue, chronic fatigue, prevalence, general population, global population, systematic review, meta-analysis

## Abstract

**Background:**

Fatigue is one of the most common subjective symptoms that impairs daily life and predict health-related events. This study aimed to estimate the prevalence of fatigue in the global population.

**Methods:**

PubMed and the Cochrane Library were used to search for relevant articles from inception to December 31, 2021. Studies with prevalence data of fatigue in the general population were selected and reviewed by three authors independently and cross-checked. Regarding subgroups, adults (≥18 years), minors (<18 years), and specific occupation population (participants in each study being limited to a specific occupational group), and fatigue types and severity, meta-analysis was conducted to produce point estimates and 95% confidence intervals (95% CI).

**Results:**

From the initial 3,432 studies, 91 studies accounting for 115 prevalence data points (623,624 participants) were finally selected. The prevalence of general fatigue (fatigue lasting < 6 months, or fatigue of unspecified duration) was 20.4% (95% CI, 16.7–25.0) in adults, 11.7% (95% CI, 5.2–26.6) in minors, and 42.3% (95% CI, 33.0–54.2) in specific occupations. Chronic fatigue (fatigue lasting more than 6 months) affected 10.1% (95% CI, 8.2–12.5) of adults, 1.5% (95% CI, 0.5–4.7) of minors, and 5.5% (95% CI, 1.4–21.6) of subjects in specific occupations. There was an overall female-predominant prevalence for all subgroup analyses, with a total odds ratio of 1.4 (95% CI, 1.3–1.6). Regarding the severity and presence of medical causes, the total prevalence of moderate fatigue [14.6% (95% CI, 9.8–21.8)] was 2.4-fold that of severe fatigue [6.1% (95% CI, 3.4–11.0)], while unexplained fatigue (fatigue experienced by individuals without any underlying medical condition that can explain the fatigue) was ~2.7-fold that of explained fatigue (fatigue experienced by individuals with a medical condition that can explain the fatigue); as proportion of 40.0% of physical, 8.6% of mental, and 28.4% of mixed cause.

**Conclusions:**

This study has produced the first comprehensive picture of global fatigue prevalence in the general population, which will provide vital reference data contributing to fatigue-related research, including the prevention of diseases.

**Systematic review registration:**

Identifier: CRD42021270498.

## Background

Fatigue refers to subjective symptoms including tiredness, weakness, lack of energy, and/or inability to concentrate (1). Fatigue can be a physiological response to stressful conditions that disappears after resting (2). However, uncontrolled fatigue (fatigue not relieved with rest), especially chronic fatigue, is a medical issue that impairs health-related quality of life (3) and productivity (4). Fatigue has been demonstrated to have significant economic implications for society (5), with an estimated cost of £1906 per chronic fatigue and chronic for fatigue syndrome (CFS) patient (mean cost for 3 months) in the United Kingdom (6). CFS represents the most severe manifestation within the spectrum of chronic fatigue-related disorders, which is characterized by core symptoms including unrefreshing sleep, post-exertional malaise (PEM), and cognitive dysfunction persisting for a duration of over six months. The economic impact of CFS on patients in the United States has reached a staggering $11,780 per year per patient (7).

In practice, fatigue is one of the top five most frequently presented health complaints in primary care (8) and can be classified by a sustained period or the presence of medical causes (9). Although fatigue is one of the most prevalent complaints in subjects suffering certain diseases, likely prevalence rate 49% in cancer patients (including both undergoing and after treatment) (10), it is also common among people without specific diagnosis (11). Furthermore, fatigue itself may indicate the development of diseases, including cancers (12). A prospective observational cohort study found that 46.9% of adults with a new episode of fatigue were diagnosed with one or more disorders in a year (13). Fatigue in the general population is also related to an increased risk of mortality (odd ratio = 2.14) (14).

Therefore, early assessment of fatigue and proper care can reduce health-related risks and economic costs. To implement proper clinical management for subjects with fatigue, determining epidemiological features, particularly prevalence, is necessary. To date, many studies showed great differences of fatigue prevalence from 4.9% (15) to 67.9% (16) among the general populations. In general, fatigue prevalence can be affected by sex, age, economic status, cultural differences and ethnicity (17–20). Thus far, most systematic reviews of fatigue prevalence have mainly focused on patients with certain diseases (21–23) or CFS (24–26), but to our knowledge, no study has been conducted in the general population.

This study aimed to create a comprehensive overview of the global prevalence and clinical features related to severity and cause of fatigue in general population.

## Methods

### Study design

To study the epidemiological features of fatigue in the general population worldwide, we systematically reviewed and analyzed fatigue-related data using public databases. This study was conducted according to the International Prospective Register of Systematic Reviews (PROSPERO) after registration (Registration # CRD42021270498).

### Data sources and keywords

This study included a search of two databases, PubMed and the Cochrane Library from inception through December 31, 2021. The search keywords were “fatigue” and “prevalence” [MeSH term]. The search terms were “(fatigue[Title]) AND ((Prevalence[Title/Abstract]) OR (Frequency[Title/Abstract]))” in PubMed, while “fatigue[Record Title] AND prevalence[Title Abstract Keyword]” and “fatigue[Record Title] AND frequency[Title Abstract Keyword]” in the Cochrane Library. All languages were included.

### Eligibility criteria

Studies were screened using the following inclusion criteria: (1) studies investigating prevalence of fatigue and (2) subjects from the general population or healthy control groups that did not have specific diseases. The exclusion criteria were as follows: (1) lifetime prevalence of fatigue, (2) fatigue measured after any interventions, (3) studies on only emotional or compassion fatigue, (4) a small number of participants (having fewer than 300 adults and minors, and < 100 for specific occupations), and (5) review studies.

### Review process and data extraction

First, three authors performed a search and screened all titles and abstracts retrieved. Based on the inclusion criteria, the full texts of the eligible studies were independently reviewed by three authors. All data were cross-checked, and further discussion was conducted with the corresponding author (Son CG) in cases of disagreement to decide. Author contact was attempted to obtain missing data. To assess the quality of the included studies, we employed the Newcastle-Ottawa Scale (NOS), which is commonly utilized in observational studies. We considered studies with a score of 7 or higher to be of high quality (27, 28). The data extracted from each study were as follows: characteristics of participants (total number, age and sex), description and number of fatigue cases, severity or medical cause information for fatigue, study design (cross-sectional/longitudinal), data collection method, fatigue assessment tool and cutoff score used, study period, publication year, and country where study was conducted. The types of fatigue were classified as general or chronic, and each prevalence was recorded. This process was determined by the consensus of researchers through discussion. The definition of fatigue subtypes (encompassing both moderate/severe fatigue and explained/unexplained fatigue) followed the different criteria as chosen by the respective authors or researchers in their original articles.

### Data coding and synthesis

The data from each study were subgrouped as follows: characteristics of participants (adults, ≥18 years; minors, < 18 years population; specific occupation), fatigue types (general, chronic), severity of fatigue (moderate, severe), medical cause of fatigue (physical, mental, mixed, drug-induced), data collection method (questionnaire, interview, telephone survey, physician reports), fatigue assessment tool (Chalder Fatigue Questionnaire, Checklist Individual Strength, Clinical Interview Schedule, Fatigue Severity Scale, Self-designed tool, Others), study year (before 2000, 2001–2010, 2011–2020), and continent where study was conducted (Europe, America, Asia, Others).

We categorized fatigue into two primary types: general fatigue, encompassing fatigue lasting < 6 months or fatigue of unspecified duration, and chronic fatigue, which denotes fatigue persisting for more than 6 months. To produce an overall characteristics fatigue prevalence, we employed a hierarchical approach. Therefore, concerning the prevalence of chronic fatigue syndrome (CFS) or CFS-like conditions, we specifically considered studies that provided simultaneous reports on the prevalence of CFS or CFS-like conditions, alongside chronic fatigue. However, we excluded prevalence data that solely focused on CFS or CFS-like illnesses.

Total fatigue includes general and chronic fatigue, while CFS-prevalence were included in that of chronic fatigue. Moreover, if articles contained data for severity-related prevalence, we further conducted binary classification (moderate or severe). We ignored the data for “mild or no” in cases of three stages (mild or no/moderate/severe fatigue).

To avoid duplicate or missing data, if fatigue prevalence was measured over multiple follow-up periods for the same participants, only the first one was included in the data. When several prevalence rates were presented with overlapping participants according to the different definitions of fatigue within a study, the prevalence defined in the broadest sense was used. Otherwise, when a study contained several prevalence rates that did not overlap, the prevalence was calculated by adding the number of participants for each definition. Regarding the analysis of the study year, the midpoint between the start and the end of the study period was used, and in case of no description, 1 year before the publication year was used.

### Statistical analysis and meta-analysis

We performed a meta-analysis using a random-effects model to produce point estimates and 95% confidence intervals (95% CI) of fatigue prevalence with subgroup analysis. The reported prevalence from each study underwent a log transformation to improve statistical properties, and pooled estimates were then back-transformed into the original prevalence scale. To account for the potentially high interstudy heterogeneity, the pooled outcome measures and their corresponding 95% CI were calculated using a random-effects model fitted with the restricted maximum likelihood estimator. The *I*^2^ statistic was used to evaluate the degree of heterogeneity between studies. All analyses were conducted using the “meta” package (by Guido Schwarzer) in R version 4.2.1. Statistical significance was determined by a hypothesis test for the analysis of differences between groups. In all analyses, *p* < 0.05 indicated statistical significance.

## Results

### Characteristics of the included studies

**Figure 1:**
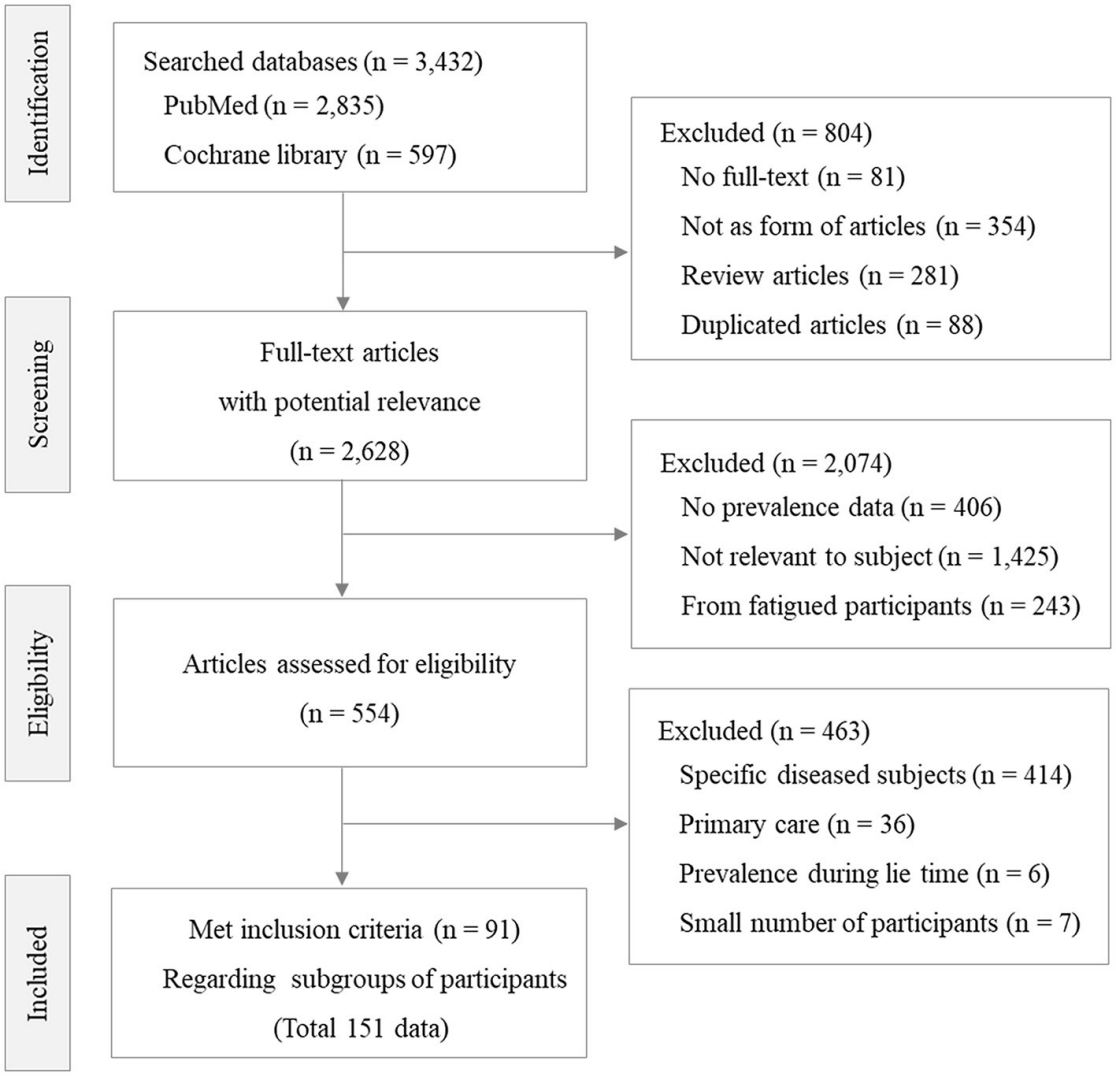
Flow diagram.

Of the initial 554 studies relevant to our study question, 91 studies (86 cross-sectional; 5 longitudinal) finally met the inclusion criteria and contained a total of 115 prevalence data points (76 general fatigue; 39 chronic fatigue; Figure 1, Supplementary Table 4). According to the results of the quality assessment, 54% (49 studies) were categorized as high quality, while 46% (42 studies) were classified as medium quality. These studies were conducted in 32 different countries beginning with a report from Finland in 1989 (29). The total number of participants was 623,624 (mean ± SD, 5,423 ± 10,992; 547,057 adults, 58,019 minors, and 18,548 specific occupation population, which included 16 occupations). Within the 115 data points, 51 data points had sex information (males 157,220; females 157,971). Thirteen data points had information on fatigue severity (moderate/severe), while 13 data points had information on the medical cause of fatigue (Table 1).

**Table 1:** Characteristics of the included studies.

**Group**		**Adults (≥18 years)**	**Minors (< 18 years)**	**Specific occupation**	**Total**
Total *n*. of included studies		55	8	28	91
Cross-sectional/longitudinal		52/3	6/2	28/0	86/5
Total *n*. of prevalence data		71	15	29	115
Total *n*. of participants		547,057	58,019	18,548	623,624
Mean *n*. of participants ± SD		(7,705 ± 13,379)	(3,868± 3,405)	(640 ± 631)	(5,423 ± 10,992)
Data with gender information		34	6	11	51
Participants *n* (M:F ratio)		288,842 (50:50)	21,696 (49:51)	4,653 (48:52)	315,191 (50:50)
***N*** **. of prevalence data by subgroup (** ***N*** **. of participants)**					
Type^a^	General fatigue	40 (409,251)	10 (36,525)	26 (15,460)	76 (461,236)
	CFS/CFS-like^b^	9 (84,530)	2 (9,428)	2 (2,445)	13 (96,403)
Severity^c^	Present	8 (51,555)	2 (1,306)	3 (1,063)	13 (53,924)
	Absent	63 (495,502)	13 (56,713)	26 (17,485)	102 (569,700)
Medical cause	Described	9 (180,938)	2 (17,172)	2 (2,445)	13 (200,555)
	Undescribed	62 (366,119)	13 (40,847)	27 (16,103)	102 (423,069)
Data collection	Questionnaire	49 (187,164)	10 (29,040)	26 (17,223)	85 (233,427)
	Interview	19 (155,996)	3 (11,807)	3 (1,325)	25 (169,128)
	Telephone survey	9 (218,277)	2 (17,172)	N/A	11 (235,449)
	Physician reports	2 (2,108)	N/A	1 (194)	3 (2,302)
Assessment tool^d^	CFQ	28 (83,496)	2 (10,603)	4 (4,284)	34 (98,383)
	CIS(a)	6 (36,567)	3 (4,773)	1 (647)	10 (41,987)
	FSS	2 (5,341)	N/A	4 (1,679)	6 (7,020)
	CIS(b)	5 (25,723)	N/A	N/A	5 (25,723)
	Self-designed tool	14 (264,219)	8 (36,592)	4 (3,239)	26 (304,050)
	Others	25 (219,198)	3 (11,807)	16 (8,699)	44 (239,704)
Continent^e^	Europe	37 (185,597)	12 (30,724)	5 (4,208)	54 (220,529)
	America	15 (262,732)	3 (27,295)	10 (5,492)	28 (295,519)
	Asia	13 (27,331)	N/A	13 (8,713)	26 (36,044)
	Others	6 (71,397)	N/A	1 (135)	7 (71,532)
Study year	Before 2000	24 (161,972)	4 (12,161)	2 (1,860)	30 (175,993)
	2001–2010	32 (259,458)	9 (34,544)	12 (5,791)	53 (299,793)
	2011–2020	15 (125,627)	2 (11,314)	15 (10,897)	32 (147,838)

^a^General fatigue represents fatigue lasting less than 6 months or fatigue of unspecified duration.

^b^Including chronic fatigue syndrome (CFS) or CFS-like data that were presented simultaneously with chronic fatigue.

^c^Studies that measured the severity of fatigue as moderate and severe were included.

^d^CFQ, Chalder Fatigue Questionnaire; CIS(a), Checklist Individual Strength; FSS, Fatigue Severity Scale; CIS(b), Clinical Interview Schedule; Self-designed tool, tools created by researchers; Others: Brief Fatigue Inventory, CDC 1988 criteria, CDC 1994 criteria, Center for Epidemiologic Studies-Depression scale, CFS Screening Questionnaire, CFS Symptom Severity Questionnaire, Chronic Fatigue Scale, Composite International Diagnostic Interview, version 3.0 (CIDI), Development and Well-being Assessment, Diagnostic Interview Schedule, Emotional State Questionnaire, Epworth Sleepiness Scores, Fatigue Assessment Scale, Fatigue Questionnaire by Japan Association of Industrial Health, Fatigue Questionnaire (30), Fatigue Scale-14, General Health Questionnaire-12, ICD-10 criteria, InterRAI-HC assessment, Iowa Fatigue Scale, Maslach Burnout Inventory, Multidimensional Assessment of Fatigue, Pediatric Screening Questionnaire, Piper Fatigue Scale, Fatigue Pictogram, Schedule of Fatigue and Anergia, Short Form Health Survey, Shortened Fatigue Questionnaire, Standard Shiftwork Index, Structured Clinical Interview for DSM-IV, Visual Analog Scale.

^e^Others: Oceania, Africa and Mixed.

Most of the studies (85 data points) collected data by questionnaire, and the remaining studies (39 data points) used interviews, telephone surveys, or physician reports. Thirty-six fatigue-assessment tools (including researcher-directed designed tools for 26 data points) were used, including the Chalder Fatigue Questionnaire (CFQ; 34 data points), Checklist Individual Strength [CIS(a); 10 data points], Fatigue Severity Scale (FSS; 6 data points), and Clinical Interview Schedule [CIS(b); 5 data points; Table 1].

### Total prevalence rate of fatigue

**Figure 2:**
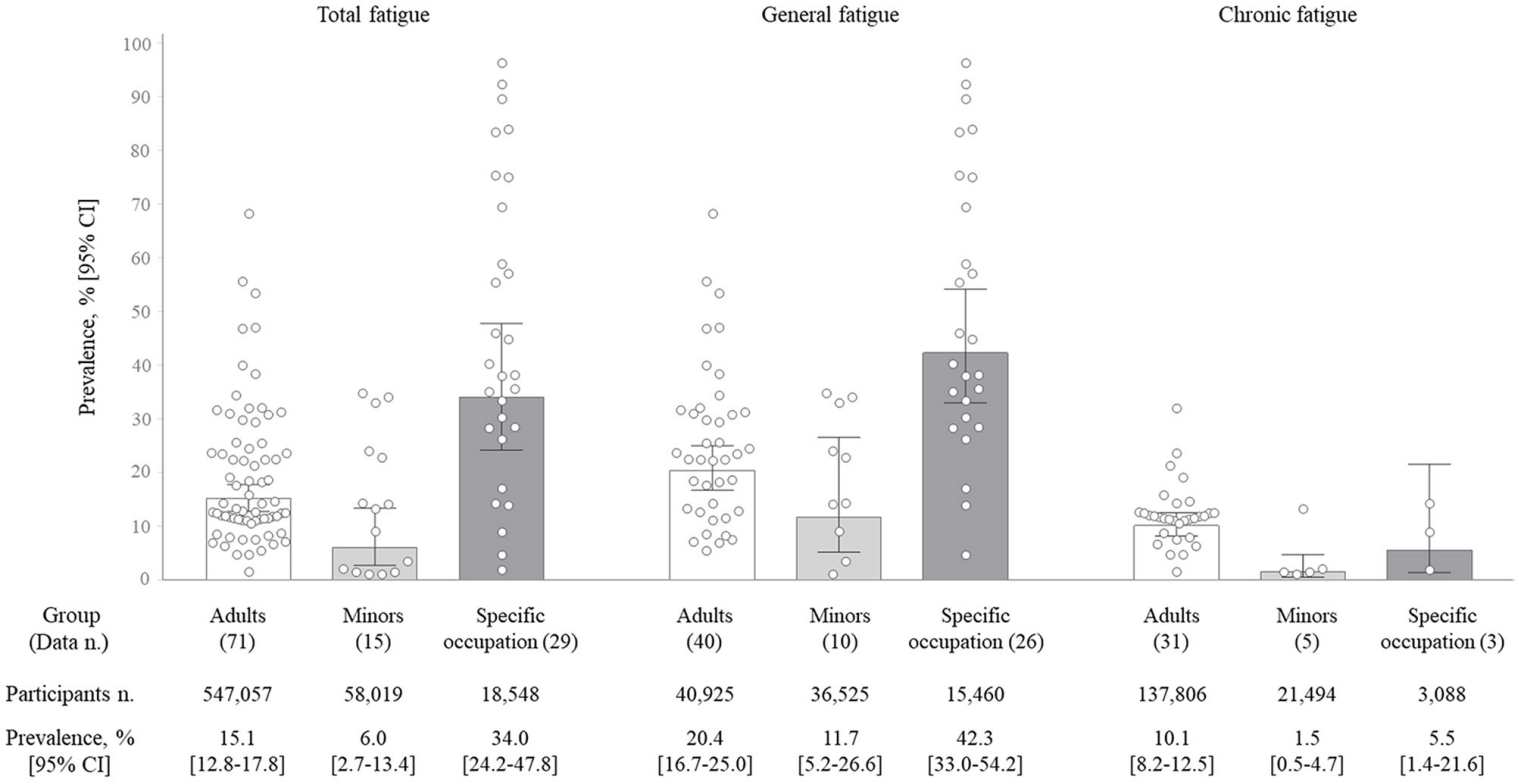
Fatigue prevalence rate by fatigue type. The bar graph shows the pooled prevalence (%), and the error bar shows the corresponding 95% confidence intervals (95% CI) by the random-effects meta-analysis model. Each dot represents the value of each study included in this analysis.

The meta-analyses showed prevalence rates of 16.4% (95% CI, 13.6–19.9) for total fatigue (115 data points), 24.2% (95% CI, 19.9–29.5) for general fatigue (76 data points), and 7.7% (95% CI, 5.7–10.3) for chronic fatigue (39 data points; Table 2). Adults showed higher fatigue prevalence rates (20.4 and 10.1% for general and chronic fatigue, respectively) than minors (11.7 and 1.5%, respectively). The specific occupation population showed prevalence rates of 42.3% (95% CI, 33.0–54.2) for general fatigue and 5.5% (95% CI, 1.4–21.6) for chronic fatigue. The differences between groups in both types of fatigue were statistically significant (*p* < 0.05; Figure 2).

**Table 2:** Fatigue prevalence by type and medical cause.

**Group**	**General**	**Chronic**	**CFS/CFS-like**	**Total**
Participants *n*. (data *n*.)	461,236 (76)	162,388 (39)	96,403 (13)	623,624 (115)
(mean ± SD)	6,069 ± 12,802	4,162 ± 6,045	7,416 ± 9,244	5,423 ± 10,992
Prevalence % [95% CI]	24.2 [19.9–29.5]	7.7 [5.7–10.3]	1.2 [0.6–2.5]	16.4 [13.6–19.9]
**Group**	**Adults (**≥**18 years)**	**Minors (**<**18 years)**	**Specific Occupation**	**Total**
Participants *n*. (data *n*.)	180,938 (9)	17,172 (2)	2,445 (2)	200,555 (13)
(mean ± SD)	20,104 ± 22,592	8,586 ± 0	1,223 ± 356	15,427 ± 19,953
Unexplained fatigue	7.6 [4.2–13.7]	0.5 [0.4–0.7]	2.0 [0.3–13.4]	4.1 [2.0–8.5]
Explained fatigue^a^	2.3 [0.6–8.6]	0.1 [0.0–0.9]	2.4 [0.2–31.4]	1.5 [0.5–4.6]
Physical cause^b^	40.0 [28.6–56.0] (3)	N/A	46.2 [20.7–100.0] (1)	40.6 [30.2–54.5] (4)
Mental cause	8.6 [6.7–11.1] (1)	N/A	N/A	8.6 [6.7–11.1] (1)
Mixed cause	28.4 [11.0–73.5] (4)	16.3 [3.4–78.3] (2)	62.9 [52.8–74.8] (1)	28.1 [14.3–55.3] (7)
Drug-induced	1.0 [0.5–2.1] (1)	N/A	N/A	1.0 [0.5–2.1] (1)

^a^Prevalence data from studies with information on the presence or absence of medical causes for reported fatigue were included, analyzed and divided into explained and unexplained fatigue.

^b^Proportion (%) of participants reporting causes of fatigue compared to total fatigue was estimated from independent data. Mixed cause included data having no description for physical and/or mental causes separately. The (number) indicates the number of data for each results.

**Figure 3:**
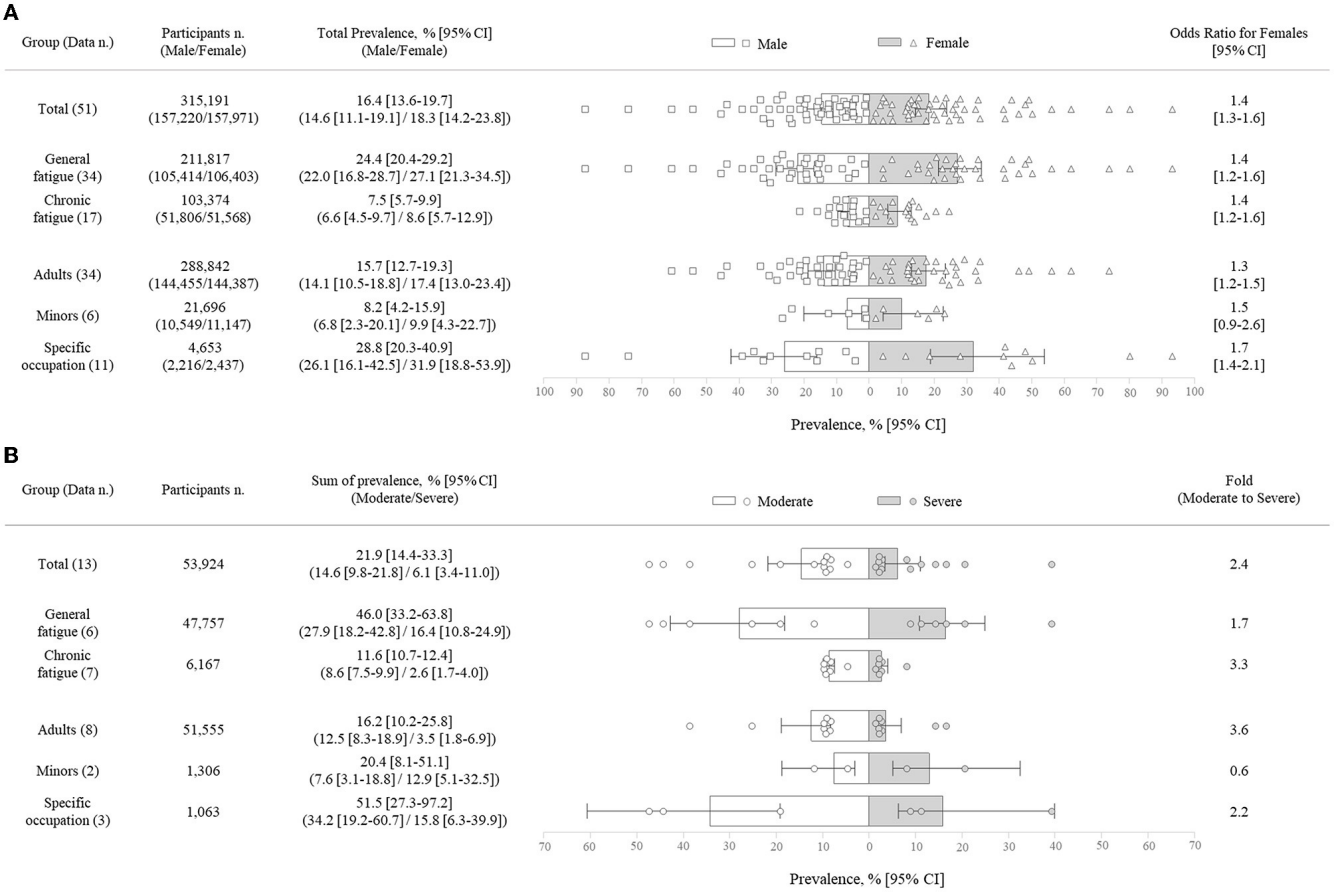
Fatigue prevalence rate by sex **(A)** and severity. **(B)** White (or gray) bar graph indicates the pooled prevalence (%) of moderate fatigue (or severe fatigue), and the error bar shows their corresponding 95% confidence intervals (95% CI) by a random-effects meta-analysis model. Each white (or gray) dot represents the value of each study included in this analysis.

Regarding sex-related fatigue prevalence (51 data points), total fatigue prevalence was 14.6% in males vs. 18.3% in females which showed female predominant (*p* = 0.23) with an odds ratio (OR) of 1.4. Prevalence rates for subgroups were 22.0% (male) vs. 27.1% (female) for general fatigue (*p* = 0.25); 6.6% (male) vs. 8.6% (female) for chronic fatigue (*p* = 0.35; Figure 3A).

### Prevalence rate by fatigue severity

Thirteen data points reported the prevalence by severity (moderate/severe). The total prevalence of moderate fatigue [14.6% (95% CI, 9.8–21.8)] was ~2.4-fold that of severe fatigue [6.1% (95% CI, 3.4–11.0); *p* < 0.05]. The ratio (moderate to severe) was ~3.6-fold in adults [12.5% (95% CI, 8.3–18.9) vs. 3.5% (95% CI, 1.8–6.9); 8 data points] and 2.2-fold in the specific occupations [34.2% (95% CI, 19.2–60.7) vs. 15.8% (95% CI, 6.3–39.9); 3 data points], while the reverse pattern (0.6-fold) was shown for minors [7.6% (95% CI, 3.1–18.8) vs. 12.9% (95% CI, 5.1–32.5); 2 data points; Figure 3B].

### Proportion of medically explained vs. unexplained fatigue

From 13 data points that reported the medical causes explaining the fatigue, the total prevalence of unexplained fatigue [4.1% (95% CI, 2.0–8.5)] was ~2.7-fold that of explained fatigue [1.5% (95% CI, 0.5–4.6); *p* = 0.13]. In adults (nine data points), the prevalence of unexplained fatigue [7.6% (95% CI, 4.2–13.7)] was 3.3-fold that of explained fatigue [2.3% (95% CI, 0.6–8.6); *p* = 0.11], while it was 5.0-fold in minors [0.5% (95% CI, 0.4–0.7) vs. 0.1% (95% CI, 0.0–0.9); 2 data points; *p* = 0.13]. In the specific occupations (2 data points), no notable difference was observed [2.0% (95% CI, 0.3–13.4) vs. 2.4% (95% CI, 0.2–31.4); *p* = 0.91; Table 2].

Regarding causes of fatigue, 40.0% were attributed to physical causes (three data points), 8.6% to mental causes (one data point), 28.4% to mixed causes (four data points), and 1.0% to drug-induced causes (one data point) in adults, while minor-derived data showed only 16.3% to mixed causes (two data points). In the specific occupations, 46.2% were attributed to physical causes (one data point) and 62.9% to mixed causes (one data point) (Table 2).

### Prevalence rate by data collection method and fatigue assessment tool

Fatigue prevalence rates were significantly different among the four data collection methods (*p* < 0.05). Questionnaire showed the highest prevalence [19.1% (95% CI, 15.7–23.3); 85 data points] followed by interviews [13.0% (95% CI, 8.1–20.7); 25 data points]. In adults, the prevalence rates were similar; interviews [15.8% (95% CI, 10.4–23.9); 19 data points] vs. questionnaires [15.0% (95% CI, 12.3–18.3); 49 data points], followed by telephone surveys [10.9% (95% CI, 6.8–17.5); 9 data points] and physician reports [8.6% (95% CI, 4.8–15.3); 2 data points; Supplementary Table 2].

Among the top four most frequently used fatigue assessment tools, the FSS showed the highest prevalence for total, followed by the CIS (a), CIS (b) and CFQ. The difference between groups was statistically significant (*p* < 0.05; Supplementary Table 2).

### Prevalence rate by continent and study year

Fatigue prevalence rates were significantly different among the continents studied (*p* < 0.05). For adults, the prevalence rate was highest in Asia [23.5% (95% CI, 13.1–42.2); 13 data points], followed by America [13.3% (95% CI, 9.5–18.7); 15 data points], and Europe [12.7% (95% CI, 10.9–14.8); 37 data points]. For the minors, the prevalence rate was highest in Europe [9.2% (95% CI, 4.1–20.9); 12 data points] and America [1.1% (95% CI, 0.4–3.0); 3 data points; Supplementary Table 3, Supplementary Figure 1].

When we compared fatigue prevalence rates by study year, there was also a significant difference between groups (*p* < 0.05). For adults, the years 2011–2020 showed the highest prevalence rates [19.8% (95% CI, 14.7–26.6); 15 data points], followed by before 2000 [15.6% (95% CI, 11.7–20.8); 24 data points] and 2001–2010 [12.9% (95% CI, 10.0–16.7); 32 data points; Supplementary Table 3].

## Discussion

Fatigue is an evolved sense in human beings to protect the body from deleterious conditions, which could occur in healthy populations (31, 32). Fatigue is frequently neglected by individuals, family members and even medical practitioners (33). However, there are reports that approximately half of the people who complain of fatigue receive one or more diagnoses within a year, including infections, anemia, thyroid dysfunction, diabetes mellitus and cancer (13). Clinically, fatigue is classified by duration, severity, or the existence of underlying disease (2, 34, 35). Commonly, chronic fatigue (≥ 6 months) presents as severe fatigue, leading to notable impairments in daily life, including poor mental health (36, 37). In the present systematic review, the average prevalence of chronic fatigue in whole data was 7.7% (95% CI, 5.7–10.3), while general fatigue (i.e., fatigue lasting < 6 months or with unspecified duration) had an average prevalence of 24.2% (95% CI, 19.9–29.5; Table 2). From the analysis for separately adults and minor population, we found that approximately a quarter and one of ten adults complain general fatigue [20.4%, (95% CI 16.7–25.0)] and chronic fatigue [11.7%, (95% CI 5.2–26.6)]. Meanwhile, one of ten or 50 adolescents presented general fatigue [10.1%, (95% 8.2–12.5)] or chronic fatigue [1.5%, (95% 0.5–4.7); Figure 2].

Fatigue appears in patients with various physical and mental diseases and is frequently not disease-specific but transdiagnostic or generic (38). Therefore, differentiating primary vs. secondary and comorbid fatigue is often a challenge (39). Nine data points indicated the predominant pattern of unexplained fatigue among adults as 3.3-fold that of explained fatigue (7.6 vs. 2.3%). Regarding the proportion of explained fatigue by medical cause, the proportion was high in the order of physical causes, mixed causes, mental causes, and drug-induced causes in total (Table 2). In general, patients with unexplained fatigue are difficult to manage in clinical care and have been reported to have a lower quality of life than those with explained fatigue (33). However, the interpretation of these results is limited because the proportion of each cause was obtained not simultaneously but individually in separate studies.

On the other hand, CFS is the most serious form of unexplained fatigue, as 52%−94% of patients are reported to work only part-time or not at all and are at greater risk of suicide (standardized mortality ratio of 6.85, compared to healthy subjects) (40, 41). No therapeutics or objective diagnostic method exists due to the unexplored etiology and pathophysiology (42). Our previous meta-analysis reported the global prevalence of CFS as 0.89% (95% CI, 0.60–1.33; 34 data points) according to CDC-1994 criteria (43). The present study calculated the proportion of CFS among subjects with chronic fatigue. From 13 data points simultaneously presenting the prevalence of both chronic fatigue and CFS (or CFS-like), 16% of chronic fatigue cases were classified as CFS(-like), which indicated a CFS(-like) prevalence of 1.2% (95% CI, 0.6–2.5) in the general population (Table 1, Supplementary Table 1). The higher prevalence of CFS in the present study than in our previous study (1.2 vs. 0.9%) might result from the inclusion of CFS-like cases in the present study. These data would be practically helpful to clinicians because unexplained chronic fatigue could be a precursor to the development of CFS (44).

Fatigue is usually recognized as a symptom cluster that accompanies other symptoms, such as pain or depression. One study reported that six out of 10 members of the general population with fatigue had pain or depression at the same time (45). These comorbid symptoms are linked to the severity of fatigue, so the guidelines for the management of fatigue recommend assessing the severity of fatigue, not just the presence of fatigue (46). In the present study, we found that moderate fatigue [14.6% (95% CI, 9.8–21.8)] accounts for 2.4-fold the prevalence of severe fatigue [6.1% (95% CI, 3.4–11.0)], similar to the pattern in most of our subgroup data, except for the minor group (1.7-fold more severe than moderate fatigue prevalence; Figure 3B). A longitudinal study showed the medical impact of severe fatigue in adolescents; 42.1% of them were diagnosed with chronic fatigue at follow-up, and they had an increased risk for the development of depression, anxiety, and CFS-related symptoms (47). The reason that the minors has more severe fatigue than moderate fatigue in present study is unclear due to the inadequate number of related studies; accordingly, our findings require caution in interpretation.

It is known that female sex and specific occupations contribute to fatigue prevalence (48, 49). Our results showed the predominance of females over males in fatigue prevalence as a 1.4 OR (95% CI, 1.3–1.6) in total and as very similar in all subgroups (Figure 3A). One proposed reason for female-predominant fatigue is an inflammatory model, rendering females more vulnerable to the detrimental effects of immune-driven behavioral changes (including fatigue, worsened mood and pain sensitivity) (50). In addition, psychosocial factors indicative of poor mental health and gender inequality can make such a difference (18, 51). As we expect, subjects working in 16 different occupations (e.g., nurses, pilots, medical workers, etc.) showed a 2.3-fold higher prevalence of total fatigue than the adult group (Figure 2). Job-related factors, including long shift hours or psychosocial work characteristics, are associated with greater fatigue (52, 53). Based on the independent risk factors for being injured in an occupational accident, fatigue in a specific occupation should be further stressed (54). Alongside gender and environmental vulnerabilities, genetic background also contributes to fatigue prevalence (55). When we analyzed data from three continents, the prevalence of fatigue in the Asian population was noticeably high, nearly twice that of the European and American populations (Supplementary Table 3, Supplementary Figure 1). Such differences across continents could be explained not only by ethnicity (56) but also by various sociodemographic features (20).

Researchers conducted many systematic reviews on fatigue prevalence, then they mainly targeted disease populations so far. This study has several limitations that need to be acknowledged. Firstly, the research was conducted using only two databases. Additionally, due to the significant heterogeneity in the fatigue measurement tools employed across studies, standardization was not feasible. Furthermore, the amount of available data for analyzing the severity and causality of fatigue was insufficient, which could impact the comprehensiveness of our conclusions. Nevertheless, to the best of our knowledge, this is the first systematic review and meta-analysis to explore the epidemiologic features of the fatigue prevalence rate in the general population worldwide.

## Conclusions

Based on the increasing health-related impact of fatigue and chronic fatigue, these results provide a valuable reference for numerous medical fields and for the prevention of diseases. The global population of 15.1% (adults) and 6.0% (minors) complain fatigue, while 10.1% of adults and 1.5% of minors are suffering from chronic fatigue, respectively. Along with 1.4-fold female-predominant prevalence, the prevalence of medically unexplained fatigue is 2.7-fold higher than explained fatigue.

## Data availability statement

The original contributions presented in the study are included in the article/Supplementary material, further inquiries can be directed to the corresponding author.

## Author contributions

J-HY, N-HP, and Y-EK had full access to all the data in the study and take responsibility for the integrity of the data and the accuracy of the data analysis. Y-CA conducted statistical analysis. J-HY and C-GS designed the study and drafted the manuscript. C-GS obtained the funding and supervised the whole process of this study. All authors have read and approved the final manuscript.
