# Supplementary figures and images for: The demographic features of fatigue in the general population worldwide: a systematic review and meta-analysis
Source: Front Public Health. 2023 Jul 28;11:1192121. doi: 10.3389/fpubh.2023.1192121 (PMC10416797; doi:10.3389/fpubh.2023.1192121)

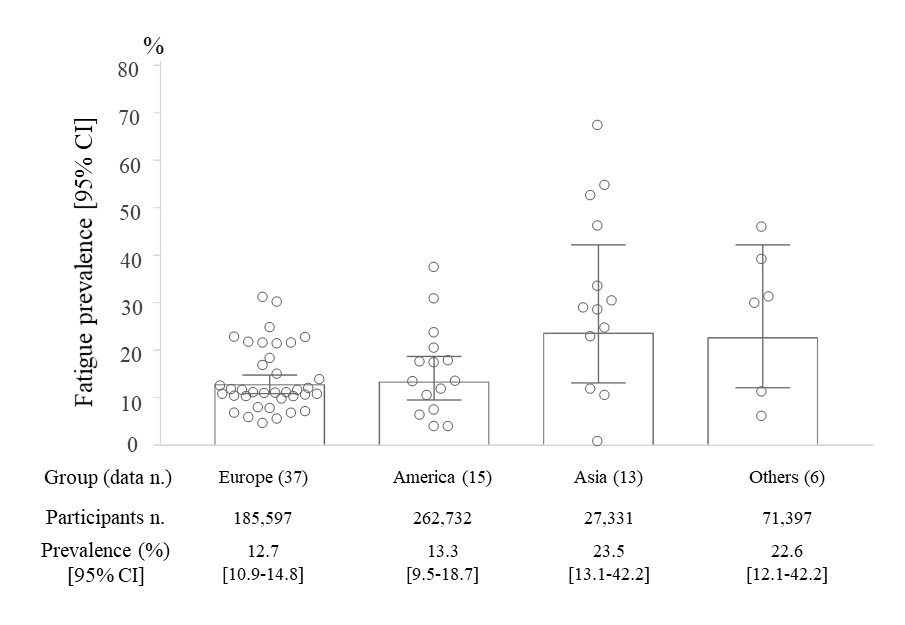

Supplement: Supplementary file 1 [file Image_1.TIFF]
