# Supplementary material for: The demographic features of fatigue in the general population worldwide: a systematic review and meta-analysis
Source: Front Public Health. 2023 Jul 28;11:1192121. doi: 10.3389/fpubh.2023.1192121 (PMC10416797; doi:10.3389/fpubh.2023.1192121)
Supplement: Supplementary file 2 [file Table_1.DOCX]

**Additional file 1**

Supplementary Table 1. Fatigue prevalence rate by fatigue type

Supplementary Table 2. Fatigue prevalence rate by data collection method and fatigue assessment tool

Supplementary Table 3. Fatigue prevalence rate by continent and study year

Supplementary Table 4. Characteristics of the 115 data points from the 91 included studies

Supplementary References

| Supplementary Table 1. Fatigue prevalence rate by fatigue type | | | | | |
| --- | --- | --- | --- | --- | --- |
| Group | | **Adults (≥18 years)** | **Minors (<18 years)** | **Specific Occupation** | **Total** |
| *General fatigue (GF)^a^, % [95% CI] (data n.)* | | | | | |
|  | Total  Male^b^  Female | 20.4 [16.7-25.0] (40)  23.5 [18.1-30.6] (19)  28.4 [22.0-36.7] (19) | 11.7 [5.2-26.6] (10)  9.7 [3.5-26.7] (5)  13.8 [7.4-25.7] (5) | 42.3 [33.0-54.2] (26)  30.5 [19.8-47.2] (10)  35.8 [21.2-60.5] (10) | 24.2 [19.9-29.5] (76)  22.0 [16.8-28.7] (34)  27.1 [21.3-34.5] (34) |
| *Chronic fatigue (CF)^c^, % [95% CI] (data n.)* | | | | | |
|  | Total  Male  Female | 10.1 [8.2-12.5] (31)  7.3 [5.1-10.5] (15)  9.4 [6.2-14.1] (15) | 1.5 [0.5-4.7] (5)  1.1 [0.8-1.6] (1)  1.8 [1.4-2.3] (1) | 5.5 [1.4-21.6] (3)  7.5 [5.4-10.4] (1)  11.0 [7.0-17.2] (1) | 7.7 [5.7-10.3] (39)  6.6 [4.5-9.7] (17)  8.6 [5.7-12.9] (17) |
| *Chronic fatigue syndrome (CFS) or CFS-like illness^d^* | | | | | |
|  | Prevalence, % [95% CI] (data n.) | 1.9 [0.8-4.3] (9) | 0.2 [0.1-0.3] (2) | 0.7 [0.5-1.2] (2) | 1.2 [0.6-2.5] (13) |
|  | Proportion of CFS(-like) in CF | 18.8% | 13.3% | 12.7% | 15.6% |
| ^a^ General fatigue represents fatigue lasting less than 6 months or fatigue of unspecified duration.  ^b^ Male and female values were based only on the data containing information on both sexes.  ^c^ Chronic fatigue represents fatigue lasting more than 6 months.  ^d^ CFS(-like) studies that presented simultaneously with chronic fatigue were included. | | | | | |

| Supplementary Table 2. Fatigue prevalence rate by data collection method and fatigue assessment tool | | | | |
| --- | --- | --- | --- | --- |
| Group | **Adults (≥18 years)** | **Minors (<18 years)** | **Specific Occupation** | **Total** |
| *Prevalence by data collection method as pooled data, % [95% CI] (data n.)* | | | | |
| Questionnaire | 15.0 [12.3-18.3] (49) | 10.5 [4.9-22.6] (10) | 38.6 [28.9-51.6] (26) | 19.1 [15.7-23.3] (85) |
| Interview | 15.8 [10.4-23.9] (19) | 3.7 [0.3-41.1] (3) | 11.3 [1.4-90.1] (3) | 13.0 [8.1-20.7] (25) |
| Telephone survey | 10.9 [6.8-17.5] (9) | 0.7 [0.4-1.1] (2) | N/A | 6.6 [3.1-14.2] (11) |
| Physician reports | 8.6 [4.8-15.3] (2) | N/A | 27.8 [21.3-36.3] (1) | 12.6 [5.5-29.2] (3) |
| *Prevalence by fatigue assessment tool^a^ as pooled data, % [95% CI] (data n.)* | | | | |
| CFQ | 10.7 [8.3-13.7] (28) | 3.5 [0.6-19.9] (2) | 26.3 [9.8-70.6] (4) | 11.1 [8.4-14.7] (34) |
| CIS(a) | 19.8 [12.2-32.1] (6) | 17.8 [9.8-32.2] (3) | 34.6 [30.4-39.5] (1) | 20.3 [14.4-28.5] (10) |
| CIS(b) | 15.8 [12.4-20.1] (5) | N/A | N/A | 15.8 [12.4-20.1] (5) |
| FSS | 26.0 [18.6-36.3] (2) | N/A | 50.7 [29.9-85.9] (4) | 40.4 [26.0-62.9] (6) |
| Self-designed tool | 21.6 [14.7-31.9] (14) | 4.5 [1.3-15.1] (8) | 64.8 [35.6-100.0] (4) | 15.8 [9.1-27.4] (26) |
| Others | 12.7 [8.8-18.3] (25) | 3.7 [0.3-41.1] (3) | 27.7 [16.5-46.6] (16) | 15.6 [11.1-21.9] (44) |
| ^a^ CFQ: Chalder Fatigue Questionnaire, CIS(a): Checklist Individual Strength, FSS: Fatigue Severity Scale, CIS(b): Clinical Interview Schedule, Self-designed tool: Tools created by researchers themselves in each study, Others: Brief Fatigue Inventory, CDC 1988 criteria, CDC 1994 criteria, Center for Epidemiologic Studies-Depression scale, CFS Screening Questionnaire, CFS Symptom Severity Questionnaire, Chronic Fatigue Scale, Composite International Diagnostic Interview, version 3.0 (CIDI), Development and Well-being Assessment, Diagnostic Interview Schedule, Emotional State Questionnaire, Epworth Sleepiness Scores, Fatigue Assessment Scale, Fatigue Questionnaire by Japan Association of Industrial Health, Fatigue Questionnaire (David et al., 1990), Fatigue Scale-14, General Health Questionnaire-12, ICD-10 criteria, InterRAI-HC assessment, Iowa Fatigue Scale, Maslach Burnout Inventory, Multidimensional Assessment of Fatigue, Pediatric Screening Questionnaire, Piper Fatigue Scale, Fatigue Pictogram, Schedule of Fatigue and Anergia, Short Form Health Survey, Shortened Fatigue Questionnaire, Standard Shiftwork Index, Structured Clinical Interview for DSM-IV, Visual Analogue Scale. | | | | |

| Supplementary Table 3. Fatigue prevalence rate by continent and study year | | | | |
| --- | --- | --- | --- | --- |
| Group | **Adults (≥18 years)** | **Minors (<18 years)** | **Specific Occupation** | **Total** |
| *Prevalence by continent as pooled data, % [95% CI] (data n.)* | | | | |
| Europe | 12.7 [10.9-14.8] (37) | 9.2 [4.1-20.9] (12) | 64.6 [45.8-91.1] (5) | 13.8 [10.9-17.6] (54) |
| America | 13.3 [9.5-18.7] (15) | 1.1 [0.4-3.0] (3) | 29.1 [15.6-54.2] (10) | 13.4 [8.5-21.3] (28) |
| Asia | 23.5 [13.1-42.2] (13) | N/A | 28.5 [16.5-49.5] (13) | 25.9 [17.4-38.4] (26) |
| Others ^a^ | 22.6 [12.1-42.2] (6) | N/A | 58.5 [46.9-73.0] (1) | 25.8 [14.3-46.6] (7) |
| *Prevalence by study year^b^ as pooled data, % [95% CI] (data n.)* | | | | |
| Before 2000 | 15.6 [11.7-20.8] (24) | 7.9 [2.5-25.2] (4) | 20.2 [9.4-43.2] (2) | 14.5 [10.9-19.3] (30) |
| 2001-2010 | 12.9 [10.0-16.7] (32) | 5.3 [1.7-16.8] (9) | 22.2 [11.3-43.8] (12) | 12.6 [9.3-17.1] (53) |
| 2011-2020 | 19.8 [14.7-26.6] (15) | 5.7 [0.2-100.0] (2) | 50.6 [38.1-67.2] (15) | 28.3 [20.4-39.3] (32) |
| ^a^ Others: Oceania, Africa and Mixed  ^b^ The midpoint between the start and the end of the study period was used as the study year, and if the study period was not provided, 1 year before the publication year was used. | | | | |

| **Supplementary Table 4. Characteristics of the 115 data points from 91 included studies** | | | | | | | |
| --- | --- | --- | --- | --- | --- | --- | --- |
| **1st author (year) Ref#** | **Fatigue type** | **Subjects** | **Participant n.**  **(male/female)** | **Data collection method** | **Fatigue assessment tool** | | **Continent** |
|  |  |  |  |  |  |  |  |
| Alavi NM et al. (2016)(1) | General | Specific | 200 | Questionnaire | Others | | Asia |
| Aljurf TM et al. (2018)(2) | General | Specific | 324 | Questionnaire | FSS | | Asia |
| Amaducci CM et al. (2010)(3) | General^a^ | Specific | 189 | Questionnaire | Others | | America |
| Anguera Md et al. (2014)(4) | General | Specific | 193 (44/149) | Questionnaire | Others | | America |
| Ball HA et al. (2010)(5) | General | ≥18 years | 4024 (1859/2165) | Questionnaire, Interview | CFQ, Others | | Asia |
| Ball HA et al. (2010)(5) | Chronic | ≥18 years | 4024 (1859/2165) | Questionnaire, Interview | CFQ, Others | | Asia |
| Baraniuk JN (2017)(6) | General | ≥18 years | 3499 (2004/1495) | Questionnaire | Others | | America |
| Beckers DG et al. (2004)(7) | General | ≥18 years | 1807 | Questionnaire | Others | | Europe |
| Bhui KS et al. (2011)(8) | Chronic | ≥18 years | 4276 (1939/2337) | Interview | CIS (b) | | Europe |
| Buchwald D et al. (1995)(9) | Chronic | ≥18 years | 3066 | Questionnaire, Telephone survey | Self-designed tool | | America |
| Bültmann U et al. (2002)(10) | General | ≥18 years | 11859 (8692/3167) | Questionnaire | CIS (a) | | Europe |
| Cai S et al. (2018)(11) | General | Specific | 1608 | Questionnaire | CFQ | | Asia |
| Camden MC et al. (2019)(12) | General | Specific | 1043 | Questionnaire | Self-designed tool | | America |
| Camden MC et al. (2019)(12) | General | Specific | 453 | Questionnaire | Self-designed tool | | America |
| Chou KL (2013)(13) | Chronic | ≥18 years | 1793 (747/1046) | Interview | CIS (b) | | Europe |
| Collin SM et al. (2015)(14) | General | <18 years | 4847 | Questionnaire | CFQ | | Europe |
| Collin SM et al. (2015)(14) | Chronic | <18 years | 5756 (2742/3014) | Questionnaire | CFQ, Self-designed tool | | Europe |
| Corfield EC et al. (2016)(15) | General | ≥18 years | 2440 (691/1749) | Questionnaire | Others | | Others |
| Crawley E et al. (2012)(16) | General | <18 years | 5657 | Questionnaire | Self-designed tool | | Europe |
| Crawley E et al. (2012)(16) | Chronic | <18 years | 5657 | Questionnaire | Self-designed tool | | Europe |
| Crowe M et al. (2017)(17) | General^a^ | ≥18 years | 45041 | Questionnaire | Others | | Others |
| de Fatima Marinho et al. (2002)(18) | Chronic | Specific | 643 (470/173) | Questionnaire | CFQ | | America |
| Dumitrescu AL et al. (2010)(19) | General | Specific | 213 (56/157) | Questionnaire | Others | | Europe |
| Eldevik MF et al. (2013)(20) | General | Specific | 1906 | Questionnaire | CFQ | | Europe |
| Evengård B et al. (2005)(21) | General^b^ | ≥18 years | 31406 | Telephone survey | Self-designed tool | | Europe |
| Evengård B et al. (2005)(21) | Chronic^b^ | ≥18 years | 31406 (16485/14921) | Telephone survey | Others | | Europe |
| Fosså SD et al. (2003)(22) | Chronic | ≥18 years | 1112 | Questionnaire | CFQ | | Europe |
| Fukuda K et al. (1997)(23) | General | ≥18 years | 1689 (748/941) | Questionnaire | Self-designed tool | | America |
| Fukuda K et al. (1997)(23) | Chronic | ≥18 years | 1689 (748/941) | Questionnaire | Self-designed tool | | America |
| Galland-Decker C et al. (2019)(24) | General | ≥18 years | 2848 (1334/1514) | Questionnaire | FSS | | Europe |
| Goedendorp MM et al. (2013)(25) | General | ≥18 years | 2300 | Questionnaire | CIS (a) | | Europe |
| Goedendorp MM et al. (2014)(26) | Chronic | ≥18 years | 1900 | Questionnaire | CIS (a) | | Europe |
| Hanevik K et al. (2014)(27) | Chronic^a^ | ≥18 years | 878 (300/578) | Questionnaire | CFQ | | Europe |
| Hanevik K et al. (2014)(27) | Chronic^a^ | ≥18 years | 1117 | Questionnaire | CFQ | | Europe |
| Hanwella R et al. (2014)(28) | General | Specific | 671 | Questionnaire | Others | | Asia |
| Hjermstad MJ et al. (2006)(29) | Chronic | ≥18 years | 2141 | Questionnaire | CFQ | | Europe |
| Hou T et al. (2020)(30) | General | Specific | 527 | Questionnaire | Others | | Asia |
| Huang LL et al. (2018)(31) | General | ≥18 years | 1579 (813/766) | Questionnaire | CFQ | | Asia |
| Hunskar GS et al. (2012)(32) | Chronic | ≥18 years | 1128 | Questionnaire | CFQ | | Europe |
| Husson O et al. (2013)(33) | General | ≥18 years | 530 | Questionnaire | Others | | Europe |
| Jackson CA et al. (2006)(34) | General | Specific | 162 (152/10) | Questionnaire | Others | | Europe |
| Jahnsen R et al. (2003)(35) | General | ≥18 years | 2323 | Questionnaire | CFQ | | Europe |
| Jahnsen R et al. (2003)(35) | Chronic | ≥18 years | 2323 | Questionnaire | CFQ | | Europe |
| Jansen NWH et al. (2003)(36) | General | ≥18 years | 6562 | Questionnaire | CIS (a) | | Europe |
| Jason LA et al. (1993)(37) | Chronic^b^ | Specific | 1474 | Questionnaire | Others | | America |
| Jason LA et al. (1999)(38) | General | ≥18 years | 18675 | Telephone survey | CFQ, Others | | America |
| Jason LA et al. (1999)(38) | Chronic^b^ | ≥18 years | 18675 (10535/8140) | Telephone survey | CFQ, Others | | America |
| Jelsness-J LP et al. (2011)(39) | General | ≥18 years | 2287 | Questionnaire | CFQ | | Europe |
| Jelsness-J LP et al. (2011)(39) | Chronic | ≥18 years | 2287 | Questionnaire | CFQ | | Europe |
| Jing MJ et al. (2015)(40) | General | ≥18 years | 1272 | Questionnaire | CFQ | | Asia |
| Jóhannsdóttir IMR et al. (2012)(41) | Chronic | ≥18 years | 755 (357/398) | Questionnaire | CFQ | | Europe |
| Jones JF et al. (2004)(42) | General^b^ | <18 years | 8586 | Telephone survey | Self-designed tool | | America |
| Jones JF et al. (2004)(42) | Chronic^b^ | <18 years | 8586 | Telephone survey | Self-designed tool | | America |
| Kang JH et al. (2009)(43) | General | Specific | 160 | Interview | FSS | | Asia |
| Kant IJ et al. (2003)(44) | General | ≥18 years | 12140 | Questionnaire | CIS (a) | | Europe |
| Kato H et al. (2019)(45) | General^a^ | Specific | 135 | Questionnaire | Others | | Others |
| Kim SH et al. (2008)(46) | Chronic^b^ | Specific | 971 | Interview | Others | | Asia |
| Lamers F et al. (2013)(47) | General | <18 years | 10123 (4953/5170) | Interview | Others | | America |
| Lawrie SM et al. (1995)(48) | General | ≥18 years | 695 (322/373) | Questionnaire | Others | | America |
| Lawrie SM et al. (1995)(48) | Chronic | ≥18 years | 695 (322/373) | Questionnaire | Others | | America |
| Lee YC et al. (2007)(49) | General | ≥18 years | 1806 (1336/470) | Questionnaire | CIS (a) | | Asia |
| Lim SM et al. (2015)(50) | General | Specific | 231 (229/2) | Questionnaire | Others | | Asia |
| Lin WQ et al. (2015)(51) | General | ≥18 years | 1158 | Questionnaire, Interview | CFQ | | Asia |
| Litleskare S et al. (2018)(52) | Chronic^a^ | ≥18 years | 696 | Questionnaire | CFQ | | Europe |
| Litleskare S et al. (2018)(52) | Chronic^a^ | ≥18 years | 852 | Questionnaire | CFQ | | Europe |
| Litleskare S et al. (2018)(52) | Chronic^a^ | ≥18 years | 843 | Questionnaire | CFQ | | Europe |
| Luntamo T et al. (2012)(53) | General | <18 years | 921 (444/477) | Questionnaire | Self-designed tool | | Europe |
| Luntamo T et al. (2012)(53) | General | <18 years | 637 (309/328) | Questionnaire | Self-designed tool | | Europe |
| Luntamo T et al. (2012)(53) | General | <18 years | 792 (383/409) | Questionnaire | Self-designed tool | | Europe |
| Martin A et al. (2007)(54) | General | ≥18 years | 2414 | Questionnaire | CFQ | | Europe |
| Martin A et al. (2007)(54) | Chronic | ≥18 years | 2414 (1147/1267) | Questionnaire | CFQ | | Europe |
| Matsuda S et al. (1997)(55) | General | Specific | 386 (201/185) | Questionnaire | Others | | Asia |
| Meng H et al. (2010)(56) | General | ≥18 years | 17084 (7534/9550) | Interview | Self-designed tool | | America |
| Moreh E et al. (2010)(57) | General | ≥18 years | 460 (187/273) | Interview | Self-designed tool | | Asia |
| Moreh E et al. (2010)(57) | General | ≥18 years | 858 (427/431) | Interview | Self-designed tool | | Asia |
| Moreh E et al. (2010)(57) | General | ≥18 years | 1162 (525/637) | Interview | Self-designed tool | | Asia |
| Narusyte J et al. (2016)(58) | Chronic | ≥18 years | 9985 | Interview | Others | | Europe |
| Nida AM et al. (2016)(59) | General | Specific | 178 | Questionnaire | Others | | America |
| Njoku MG et al. (2007)(60) | General | ≥18 years | 1054 (455/599) | Questionnaire, Interview, Physician | | CFQ, Others | Others |
| Njoku MG et al. (2007)(60) | Chronic | ≥18 years | 1054 (455/599) | Questionnaire, Interview, Physician | | CFQ, Others | Others |
| Palupi KC et al. (2017)(61) | General | Specific | 194 | Interview, Physician | Others | | Asia |
| Parsons PL et al. (2011)(62) | General | ≥18 years | 16182 | Interview | Self-designed tool | | America |
| Patel V et al. (2005)(63) | Chronic | ≥18 years | 2494 | Interview | CIS (b) | | Asia |
| Patterson AJ et al. (2000)(64) | General | ≥18 years | 20373 | Questionnaire | Self-designed tool, Others | | Others |
| Persson R et al. (2015)(65) | Chronic | ≥18 years | 847 | Questionnaire | CFQ | | Europe |
| Raftopoulos V et al. (2012)(66) | General | Specific | 1471 (282/1189) | Questionnaire | Self-designed tool | | Europe |
| Reeves WC et al. (2007)(67) | General | ≥18 years | 19381 | Telephone survey | Self-designed tool | | America |
| Reis C et al. (2013)(68) | General | Specific | 456 | Questionnaire | FSS | | Europe |
| Ricci JA et al. (2007)(69) | General^b^ | ≥18 years | 76060 (40813/35247) | Telephone survey | Self-designed tool | | America |
| Rimes KA et al. (2007)(70) | General | <18 years | 842 | Interview | Others | | Europe |
| Rimes KA et al. (2007)(70) | Chronic | <18 years | 842 | Interview | Others | | Europe |
| Shattuck NL et al. (2016)(71) | General^a^ | Specific | 739 | Questionnaire | FSS | | America |
| Steele L et al. (1998)(72) | Chronic^b^ | ≥18 years | 14607 (7198/7409) | Telephone survey | Self-designed tool, Others | | America |
| Sunwoo JS et al. (2022)(73) | General | ≥18 years | 2493 (1236/1257) | Interview | FSS | | Asia |
| Tanaka M et al. (2008)(74) | General | Specific | 127 (89/38) | Questionnaire | CFQ | | Asia |
| ter Wolbeek M et al. (2006)(75) | General | <18 years | 3467 (1718/1749) | Questionnaire | CIS (a) | | Europe |
| ter Wolbeek M et al. (2008)(76) | General^a^ | <18 years | 653 | Questionnaire | CIS (a) | | Europe |
| ter Wolbeek M et al. (2008)(76) | Chronic^a^ | <18 years | 653 | Questionnaire | CIS (a) | | Europe |
| van Deuren S et al. (2022)(77) | Chronic | ≥18 years | 978 | Questionnaire | Others | | Europe |
| van't Leven M et al. (2010)(78) | General^b^ | ≥18 years | 9062 | Questionnaire | Others | | Europe |
| van't Leven M et al. (2010)(78) | Chronic^b^ | ≥18 years | 9062 | Questionnaire | Others | | Europe |
| Vasconcelos SP et al. (2011)(79) | General | Specific | 272 (49/223) | Questionnaire | Self-designed tool | | America |
| Vestergaard S et al. (2009)(80) | General | ≥18 years | 1055 (464/591) | Questionnaire | Others | | Europe |
| Vetkas A et al. (2020)(81) | General | ≥18 years | 3923 | Questionnaire | Others | | Europe |
| Violanti JM et al. (2018)(82) | General | Specific | 308 (230/78) | Questionnaire | Others | | America |
| Walker EA et al. (1993)(83) | General^b^ | ≥18 years | 10533 | Interview | Others | | America |
| Wang FW et al. (2009)(84) | General | Specific | 647 (414/233) | Questionnaire | CIS (a) | | Asia |
| Watanabe N et al. (2008)(85) | General | ≥18 years | 8580 | Interview | CIS (b) | | Europe |
| Watanabe N et al. (2008)(85) | Chronic | ≥18 years | 8580 (3852/4728) | Interview | CIS (b) | | Europe |
| Wendt A et al. (2019)(86) | General | ≥18 years | 60202 (25920/34282) | Interview | Self-designed tool | | America |
| Wensaas KA et al. (2012)(87) | Chronic^a^ | ≥18 years | 1128 (390/738) | Questionnaire | CFQ | | Europe |
| Wong WS et al. (2010)(88) | Chronic | ≥18 years | 5001 (2260/2741) | Telephone survey | CFQ, Others | | Asia |
| Yun YH et al. (2008)(89) | General^a,b^ | ≥18 years | 1000 (501/499) | Questionnaire | Others | | Asia |
| Zarrouq B et al. (2021)(90) | General | ≥18 years | 1435 | Questionnaire | CFQ | | Others |
| Zhan YX et al. (2020)(91) | General | Specific | 2667 | Questionnaire | Others | | Asia |

**Supplementary References**

1. Alavi NM, Madani M, Sadat Z, Kashani HH, Sharif MR. Fatigue and vitamin D status in Iranian female nurses. Global journal of health science. 2016;8(6):196.

2. Aljurf TM, Olaish AH, BaHammam AS. Assessment of sleepiness, fatigue, and depression among Gulf Cooperation Council commercial airline pilots. Sleep and Breathing. 2018;22(2):411-9.

3. Amaducci CdM, Mota DDFdC, Pimenta CAdM. Fatigue among nursing undergraduate students. Revista da Escola de Enfermagem da USP. 2010;44:1052-8.

4. Anguera MdG, Gianini RJ. Prevalence of fatigue reported by physiotherapists operating diathermy equipment for microwave. Revista Brasileira de Epidemiologia. 2014;17:577-81.

5. Ball HA, Sumathipala A, Siribaddana SH, Kovas Y, Glozier N, McGuffin P, et al. Aetiology of fatigue in Sri Lanka and its overlap with depression. The British Journal of Psychiatry. 2010;197(2):106-13.

6. Baraniuk JN. Chronic fatigue syndrome prevalence is grossly overestimated using Oxford criteria compared to Centers for Disease Control (Fukuda) criteria in a US population study. Fatigue: biomedicine, health & behavior. 2017;5(4):215-30.

7. Beckers DG, van der Linden D, Smulders PG, Kompier MA, van Veldhoven MJ, van Yperen NW. Working overtime hours: relations with fatigue, work motivation, and the quality of work. Journal of Occupational and Environmental Medicine. 2004:1282-9.

8. Bhui KS, Dinos S, Ashby D, Nazroo J, Wessely S, White PD. Chronic fatigue syndrome in an ethnically diverse population: the influence of psychosocial adversity and physical inactivity. BMC medicine. 2011;9(1):1-12.

9. Buchwald D, Umali P, Umali J, Kith P, Pearlman T, Komaroff AL. Chronic fatigue and the chronic fatigue syndrome: prevalence in a Pacific Northwest health care system. Annals of internal medicine. 1995;123(2):81-8.

10. Bültmann U, Kant I, Kasl SV, Beurskens AJ, van den Brandt PA. Fatigue and psychological distress in the working population: psychometrics, prevalence, and correlates. Journal of psychosomatic research. 2002;52(6):445-52.

11. Cai S, Lin H, Hu X, Cai Y-X, Chen K, Cai W-Z. High fatigue and its associations with health and work related factors among female medical personnel at 54 hospitals in Zhuhai, China. Psychology, health & medicine. 2018;23(3):304-16.

12. Camden MC, Medina-Flintsch A, Hickman JS, Bryce J, Flintsch G, Hanowski RJ. Prevalence of operator fatigue in winter maintenance operations. Accident Analysis & Prevention. 2019;126:47-53.

13. Chou K-L. Chronic fatigue and affective disorders in older adults: Evidence from the 2007 British National Psychiatric Morbidity Survey. Journal of affective disorders. 2013;145(3):331-5.

14. Collin SM, Norris T, Nuevo R, Tilling K, Joinson C, Sterne JA, et al. Chronic fatigue syndrome at age 16 years. Pediatrics. 2016;137(2).

15. Corfield EC, Martin NG, Nyholt DR. Shared genetic factors in the co-occurrence of depression and fatigue. Twin Research and Human Genetics. 2016;19(6):610-8.

16. Crawley E, Hughes R, Northstone K, Tilling K, Emond A, Sterne JA. Chronic disabling fatigue at age 13 and association with family adversity. Pediatrics. 2012;130(1):e71-e9.

17. Crowe M, Jordan J, Gillon D, McCall C, Frampton C, Jamieson H. The prevalence of pain and its relationship to falls, fatigue, and depression in a cohort of older people living in the community. Journal of advanced nursing. 2017;73(11):2642-51.

18. de Fatima Marinho de Souza M, Messing K, Menezes P, Cho H. Chronic fatigue among bank workers in Brazil. Occupational Medicine. 2002;52(4):187-94.

19. Dumitrescu AL, Toma C, Lascu V. Associations among sleep disturbance, vitality, fatigue and oral health. Oral Health & Preventive Dentistry. 2010;8(4):323-30.

20. Eldevik MF, Flo E, Moen BE, Pallesen S, Bjorvatn B. Insomnia, excessive sleepiness, excessive fatigue, anxiety, depression and shift work disorder in nurses having less than 11 hours in-between shifts. PloS one. 2013;8(8):e70882.

21. Evengård B, Jacks A, Pedersen NL, Sullivan PF. The epidemiology of chronic fatigue in the Swedish Twin Registry. Psychological medicine. 2005;35(9):1317-26.

22. Fosså SD, Dahl AA, Loge JH. Fatigue, anxiety, and depression in long-term survivors of testicular cancer. Journal of Clinical Oncology. 2003;21(7):1249-54.

23. Fukuda K, Dobbins JG, Wilson LJ, Dunn RA, Wilcox K, Smallwood D. An epidemiologic study of fatigue with relevance for the chronic fatigue syndrome. Journal of psychiatric research. 1997;31(1):19-29.

24. Galland-Decker C, Marques-Vidal P, Vollenweider P. Prevalence and factors associated with fatigue in the Lausanne middle-aged population: a population-based, cross-sectional survey. BMJ open. 2019;9(8):e027070.

25. Goedendorp MM, Hoitsma AJ, Bloot L, Bleijenberg G, Knoop H. Severe fatigue after kidney transplantation: a highly prevalent, disabling and multifactorial symptom. Transplant International. 2013;26(10):1007-15.

26. Goedendorp MM, Tack CJ, Steggink E, Bloot L, Bazelmans E, Knoop H. Chronic fatigue in type 1 diabetes: highly prevalent but not explained by hyperglycemia or glucose variability. Diabetes care. 2014;37(1):73-80.

27. Hanevik K, Wensaas K-A, Rortveit G, Eide GE, Mørch K, Langeland N. Irritable bowel syndrome and chronic fatigue 6 years after giardia infection: a controlled prospective cohort study. Clinical infectious diseases. 2014;59(10):1394-400.

28. Hanwella R, Jayasekera N, de Silva VA. Fatigue symptoms in Sri Lanka Navy personnel deployed in combat areas. Ceylon Medical Journal. 2014;59(2).

29. Hjermstad MJ, Oldervoll L, Fosså SD, Holte H, Jacobsen AB, Loge JH. Quality of life in long-term Hodgkin’s disease survivors with chronic fatigue. European journal of cancer. 2006;42(3):327-33.

30. Hou T, Zhang R, Song X, Zhang F, Cai W, Liu Y, et al. Self-efficacy and fatigue among non-frontline health care workers during COVID-19 outbreak: A moderated mediation model of posttraumatic stress disorder symptoms and negative coping. PloS one. 2020;15(12):e0243884.

31. Huang L-L, Guo D-H, Jing M-J, Wang X-X, Liu N, Wang P-X. A correlation between sickness or injury within two weeks, chronic diseases and fatigue among adults aged 18–45 years. Psychology, Health & Medicine. 2018;23(4):434-41.

32. Hunskar GS, Langeland N, Wensaas K-A, Hanevik K, Eide GE, Mørch K, et al. The impact of atopic disease on the risk of post-infectious fatigue and irritable bowel syndrome 3 years after Giardia infection. A historic cohort study. Scandinavian journal of gastroenterology. 2012;47(8-9):956-61.

33. Husson O, Nieuwlaat W-A, Oranje WA, Haak HR, van de Poll-Franse LV, Mols F. Fatigue among short-and long-term thyroid cancer survivors: results from the population-based PROFILES registry. Thyroid. 2013;23(10):1247-55.

34. Jackson CA, Earl L. Prevalence of fatigue among commercial pilots. Occupational medicine. 2006;56(4):263-8.

35. Jahnsen R, Villien L, Stanghelle JK, Holm I. Fatigue in adults with cerebral palsy in Norway compared with the general population. Developmental Medicine and Child Neurology. 2003;45(5):296-303.

36. Jansen N, Van Amelsvoort L, Kristensen T, Van den Brandt P, Kant I. Work schedules and fatigue: a prospective cohort study. Occupational and environmental medicine. 2003;60(suppl 1):i47-i53.

37. Jason LA, Taylor SL, Johnson S, Goldston SE, Salina D, Bishop P, et al. Prevalence of chronic fatigue syndrome-related symptoms among nurses. Evaluation & the Health Professions. 1993;16(4):385-99.

38. Jason LA, Richman JA, Rademaker AW, Jordan KM, Plioplys AV, Taylor RR, et al. A community-based study of chronic fatigue syndrome. Archives of internal medicine. 1999;159(18):2129-37.

39. Jelsness-Jørgensen L-P, Bernklev T, Henriksen M, Torp R, Moum BA. Chronic fatigue is more prevalent in patients with inflammatory bowel disease than in healthy controls. Inflammatory bowel diseases. 2011;17(7):1564-72.

40. Jing M-J, Wang J-J, Lin W-Q, Lei Y-X, Wang P-X. A community-based cross-sectional study of fatigue in middle-aged and elderly women. Journal of psychosomatic research. 2015;79(4):288-94.

41. Jóhannsdóttir IM, Hjermstad MJ, Moum T, Wesenberg F, Hjorth L, Schrøder H, et al. Increased prevalence of chronic fatigue among survivors of childhood cancers: a population‐based study. Pediatric blood & cancer. 2012;58(3):415-20.

42. Jones JF, Nisenbaum R, Solomon L, Reyes M, Reeves WC. Chronic fatigue syndrome and other fatiguing illnesses in adolescents: a population-based study. Journal of Adolescent Health. 2004;35(1):34-40.

43. Kang J-H, Chen S-C. Effects of an irregular bedtime schedule on sleep quality, daytime sleepiness, and fatigue among university students in Taiwan. BMC public health. 2009;9(1):1-6.

44. Kant I, Bültmann U, Schröer K, Beurskens A, Van Amelsvoort L, Swaen G. An epidemiological approach to study fatigue in the working population: the Maastricht Cohort Study. Occupational and environmental medicine. 2003;60(suppl 1):i32-i9.

45. Kato H, Burger AP, Emoto K, Sakama R, Uehara Y, Segon A, et al. Prevalence of fatigue among postgraduate trainees in the United States and Japan. Journal of general and family medicine. 2019;20(6):260-3.

46. Kim S-H, Lee K, Lim H-S. Prevalence of chronic widespread pain and chronic fatigue syndrome in Korean livestock raisers. Journal of occupational health. 2008:0810200036-.

47. Lamers F, Hickie I, Merikangas KR. Prevalence and correlates of prolonged fatigue in a US sample of adolescents. American journal of psychiatry. 2013;170(5):502-10.

48. Lawrie S, Pelosi A. Chronic fatigue syndrome in the community prevalence and associations. The British Journal of Psychiatry. 1995;166(6):793-7.

49. Lee Y-C, Chien K-L, Chen H-H. Lifestyle risk factors associated with fatigue in graduate students. Journal of the Formosan Medical Association. 2007;106(7):565-72.

50. Lim SM, Chia SE. The prevalence of fatigue and associated health and safety risk factors among taxi drivers in Singapore. Singapore medical journal. 2015;56(2):92.

51. Lin W-Q, Jing M-J, Tang J, Wang J-J, Zhang H-S, Yuan L-X, et al. Factors associated with fatigue among men aged 45 and older: A cross-sectional study. International journal of environmental research and public health. 2015;12(9):10897-909.

52. Litleskare S, Rortveit G, Eide GE, Hanevik K, Langeland N, Wensaas K-A. Prevalence of irritable bowel syndrome and chronic fatigue 10 years after Giardia infection. Clinical Gastroenterology and Hepatology. 2018;16(7):1064-72. e4.

53. Luntamo T, Sourander A, Santalahti P, Aromaa M, Helenius H. Prevalence changes of pain, sleep problems and fatigue among 8-year-old children: years 1989, 1999, and 2005. Journal of Pediatric Psychology. 2012;37(3):307-18.

54. Martin A, Chalder T, Rief W, Braehler E. The relationship between chronic fatigue and somatization syndrome: a general population survey. Journal of psychosomatic research. 2007;63(2):147-56.

55. Matsuda S, LUONG NA, THUNG DH, van TRINH L, HIEN HM, DAT PH, et al. A study of complaints of fatigue by workers employed in Vietnamese factories with newly imported technology. Industrial health. 1997;35(1):16-28.

56. Meng H, Hale L, Friedberg F. Prevalence and predictors of fatigue among middle-aged and older adults: evidence from the Health and Retirement study. Journal of the American Geriatrics Society. 2010;58(10):2033.

57. Moreh E, Jacobs JM, Stessman J. Fatigue, function, and mortality in older adults. Journals of Gerontology Series A: Biomedical Sciences and Medical Sciences. 2010;65(8):887-95.

58. Narusyte J, Ropponen A, Alexanderson K, Svedberg P. Genetic and environmental influences on disability pension due to mental diagnoses: limited importance of major depression, generalized anxiety, and chronic fatigue. Twin Research and Human Genetics. 2016;19(1):10-6.

59. Nida AM, Googe BJ, Lewis AF, May WL. Resident fatigue in otolaryngology residents: a Web based survey. American Journal of Otolaryngology. 2016;37(3):210-6.

60. Njoku MGC, Jason LA, Torres-Harding SR. The prevalence of chronic fatigue syndrome in Nigeria. Journal of health psychology. 2007;12(3):461-74.

61. Palupi KC, Shih C-K, Chang J-S. Cooking methods and depressive symptoms are joint risk factors for fatigue among migrant Indonesian women working domestically in Taiwan. Asia Pacific journal of clinical nutrition. 2017;26(Supplement).

62. Parsons PL, Mezuk B, Ratliff S, Lapane KL. Subsidized housing not subsidized health: health status and fatigue among elders in public housing and other community settings. Ethnicity & disease. 2011;21(1):85.

63. Patel V, Kirkwood BR, Weiss H, Pednekar S, Fernandes J, Pereira B, et al. Chronic fatigue in developing countries: population based survey of women in India. Bmj. 2005;330(7501):1190.

64. Patterson AJ, Brown WJ, Powers JR, Roberts DC. Iron deficiency, general health and fatigue: results from the Australian Longitudinal Study on Women's Health. Quality of Life Research. 2000;9(5):491-7.

65. Persson R, Wensaas K-A, Hanevik K, Eide GE, Langeland N, Rortveit G. The relationship between irritable bowel syndrome, functional dyspepsia, chronic fatigue and overactive bladder syndrome: a controlled study 6 years after acute gastrointestinal infection. BMC gastroenterology. 2015;15(1):1-7.

66. Raftopoulos V, Charalambous A, Talias M. The factors associated with the burnout syndrome and fatigue in Cypriot nurses: a census report. BMC public health. 2012;12(1):1-13.

67. Reeves WC, Jones JF, Maloney E, Heim C, Hoaglin DC, Boneva RS, et al. Prevalence of chronic fatigue syndrome in metropolitan, urban, and rural Georgia. Population health metrics. 2007;5(1):1-10.

68. Reis C, Mestre C, Canhão H. Prevalence of fatigue in a group of airline pilots. Aviation, space, and environmental medicine. 2013;84(8):828-33.

69. Ricci JA, Chee E, Lorandeau AL, Berger J. Fatigue in the US workforce: prevalence and implications for lost productive work time. Journal of occupational and environmental medicine. 2007:1-10.

70. Rimes KA, Goodman R, Hotopf M, Wessely S, Meltzer H, Chalder T. Incidence, prognosis, and risk factors for fatigue and chronic fatigue syndrome in adolescents: a prospective community study. Pediatrics. 2007;119(3):e603-e9.

71. Shattuck NL, Matsangas P, Moore J, Wegemann L. Prevalence of musculoskeletal symptoms, excessive daytime sleepiness, and fatigue in the crewmembers of a US Navy ship. Military medicine. 2016;181(7):655-62.

72. Steele L, Dobbins JG, Fukuda K, Reyes M, Randall B, Koppelman M, et al. The epidemiology of chronic fatigue in San Francisco. The American journal of medicine. 1998;105(3):83S-90S.

73. Sunwoo J-S, Kim D, Chu MK, Yun C-H, Yang KI. Fatigue is associated with depression independent of excessive daytime sleepiness in the general population. Sleep and Breathing. 2022;26(2):933-40.

74. Tanaka M, Mizuno K, Fukuda S, Shigihara Y, Watanabe Y. Relationships between dietary habits and the prevalence of fatigue in medical students. Nutrition. 2008;24(10):985-9.

75. Ter Wolbeek M, Van Doornen LJ, Kavelaars A, Heijnen CJ. Severe fatigue in adolescents: a common phenomenon? Pediatrics. 2006;117(6):e1078-e86.

76. ter Wolbeek M, van Doornen LJ, Kavelaars A, Heijnen CJ. Predictors of persistent and new-onset fatigue in adolescent girls. Pediatrics. 2008;121(3):e449-e57.

77. van Deuren S, Penson A, van Dulmen‐den Broeder E, Grootenhuis MA, van der Heiden‐van der Loo M, Bronkhorst E, et al. Prevalence and risk factors of cancer‐related fatigue in childhood cancer survivors: A DCCSS LATER study. Cancer. 2022;128(5):1110-21.

78. van’t Leven M, Zielhuis GA, van der Meer JW, Verbeek AL, Bleijenberg G. Fatigue and chronic fatigue syndrome-like complaints in the general population. European journal of public health. 2010;20(3):251-7.

79. Vasconcelos SP, Fischer FM, Reis AOA, Moreno CRdC. Factors associated with work ability and perception of fatigue among nursing personnel from Amazonia. Revista Brasileira de Epidemiologia. 2011;14:688-97.

80. Vestergaard S, Nayfield SG, Patel KV, Eldadah B, Cesari M, Ferrucci L, et al. Fatigue in a representative population of older persons and its association with functional impairment, functional limitation, and disability. Journals of Gerontology Series A: Biomedical Sciences and Medical Sciences. 2009;64(1):76-82.

81. Vetkas A, Prans E, Kõks S, Rätsep T, Asser T. Aneurysmal subarachnoid haemorrhage: effect of CRHR1 genotype on fatigue and depression. BMC neurology. 2020;20(1):1-9.

82. Violanti JM, Owens SL, Fekedulegn D, Ma CC, Charles LE, Andrew ME. An exploration of shift work, fatigue, and gender among police officers: the BCOPS study. Workplace health & safety. 2018;66(11):530-7.

83. Walker EA, Katon WJ, Jemelka RP. Psychiatric disorders and medical care utilization among people in the general population who report fatigue. Journal of General Internal Medicine. 1993;8(8):436-40.

84. Wang F-W, Chiu Y-W, Tu M-S, Chou M-Y, Wang C-L, Chuang H-Y. Chronic fatigue of the small enterprise workers participating in an occupational health checkup center in southern Taiwan. International archives of occupational and environmental health. 2009;82(7):819-25.

85. Watanabe N, Stewart R, Jenkins R, Bhugra DK, Furukawa TA. The epidemiology of chronic fatigue, physical illness, and symptoms of common mental disorders: a cross-sectional survey from the second British National Survey of Psychiatric Morbidity. Journal of psychosomatic research. 2008;64(4):357-62.

86. Wendt A, Costa CS, Machado AKF, Costa FS, Neves RG, Flores TR, et al. Sleep disturbances and daytime fatigue: data from the Brazilian National Health Survey, 2013. Cadernos de Saúde Pública. 2019;35.

87. Wensaas K-A, Langeland N, Hanevik K, Mørch K, Eide GE, Rortveit G. Irritable bowel syndrome and chronic fatigue 3 years after acute giardiasis: historic cohort study. Gut. 2012;61(2):214-9.

88. Wong WS, Fielding R. Prevalence of chronic fatigue among Chinese adults in Hong Kong: a population-based study. Journal of affective disorders. 2010;127(1-3):248-56.

89. Yun YH, Lee MK, Chun HN, Lee YM, Park SM, Mendoza TR, et al. Fatigue in the general Korean population: application and normative data of the Brief Fatigue Inventory. Journal of pain and symptom management. 2008;36(3):259-67.

90. Zarrouq B, Abbas N, Hilaly JE, Asri AE, Abbouyi S, Omari M, et al. An investigation of the association between religious coping, fatigue, anxiety and depressive symptoms during the COVID-19 pandemic in Morocco: a web-based cross-sectional survey. BMC psychiatry. 2021;21(1):1-13.

91. Zhan Y-x, Zhao S-y, Yuan J, Liu H, Liu Y-f, Gui L-l, et al. Prevalence and influencing factors on fatigue of first-line nurses combating with COVID-19 in China: a descriptive cross-sectional study. Current medical science. 2020;40(4):625-35.
